# Gray Wolf Diet Composition in California’s Human-Dominated Landscape

**DOI:** 10.1371/journal.pone.0351768

**Published:** 2026-07-08

**Authors:** Tina L. Saitone, Kenneth W. Tate, Benjamin J. Sacks

**Affiliations:** 1 Department of Agricultural and Resource Economics, University of California, Davis, California, United States of America; 2 Department of Plant Sciences, University of California, Davis, California, United States of America; 3 Department of Population Health and Reproduction, Veterinary Genetics Laboratory, University of California, Davis, California, United States of America; Zoological Survey of India, INDIA

## Abstract

Gray wolves have successfully recolonized much of their historical range on multiple continents, now occupying landscapes substantially altered during their absence. Wolf ecological function in human-modified landscapes remains poorly understood, fueling conflict concerns among rural stakeholders and conservation advocates over predation on economically valuable livestock and wildlife. Dietary studies can facilitate prediction of ecological impacts and help avoid outcomes that undermine human-wolf coexistence and conservation objectives. This study addresses a critical gap in dietary research of a recolonizing population of wolves in California, USA, representing the first investigation employing genetic identification of species and individuals combined with DNA metabarcoding for diet determination in scat samples. Our findings indicate that cattle dominated wolf diet, occurring in 72% of samples during the study period, compared to mule deer, which occurred in 45% of samples and small mammals that appeared in 51% of samples. Dietary biomass estimates corroborated cattle as the principal dietary component accounting for an average of 55%, while mule deer and small mammals each contributed considerably smaller proportions, 12% and 15%, respectively. These results revealed that wolves in California’s human-modified landscape primarily satisfy their caloric requirements through livestock consumption, underscoring the considerable challenges posed by coexistence of wolves and humans within the state.

## Introduction

Gray wolves (*Canis lupus*) once inhabited nearly the entire northern hemisphere, spanning Europe, Asia, and North America [1]. In the United States, the species occupied more than two-thirds of the country’s landmass [2]. Systematic extirpation resulted in their near-complete elimination from the contiguous United States by the mid-1900s. With the aid of legal protections (e.g., U.S. Endangered Species Act) and reintroduction programs, gray wolves have earned the distinction of one of the most successful species in reestablishing itself across its historic range [3]. Wolves are now occupying landscapes that have been significantly altered by human activity during the intervening decades or centuries of their absence [3]. The ecological role of wolves in human-modified landscapes remains highly debated and poorly understood, leading to significant concern among rural communities and stakeholders who anticipate, or are experiencing, increased conflict from wolf predation on livestock and wildlife species that have both economic and intrinsic value to humans [4,5]. Nowhere is this more evident than in California.

Almost two decades after gray wolves were reintroduced to central Idaho and Yellowstone National Park, their descendants had expanded and recolonized much of the Pacific Northwest, with the first confirmed wolf entering California in late 2011, following a nearly 100-year absence [6]. The Lassen Pack – identified in 2016 as the first breeding pair to establish a lasting presence in California – marked the beginning of wolf population growth and expansion of geographic extent in the State [6]. By the end of 2024, California was home to at least seven packs, and the year-end minimum count of individuals totaled 50 [6]. Although wolf populations in California have expanded substantially in both numbers and distribution, gray wolves remain listed under both the U.S. Endangered Species Act and the California Endangered Species Act [7].

Existing research indicates that under natural conditions, gray wolves typically shift to alternative prey when populations of their primary wild ungulate prey decline [8]. However, in human-modified landscapes across the globe, some gray wolf populations are documented to consume substantial quantities of anthropogenic food, in cases when wild prey remain abundant (e.g., [9–11]). In other parts of the world where wild ungulates are nearly absent, gray wolves have acclimated to densely populated and heavily human-modified areas by primarily subsisting on alternative food sources and livestock [12]. These behavioral modifications illustrate the opportunistic foraging strategy of wolves and their inclination toward food sources that require less energy to obtain and pose lower perceived risk of injury during acquisition and consumption [13].

Despite documented regular, and often significant, use of anthropogenic food by wolves, this facet of their foraging ecology and its broader ramifications have received surprisingly limited scientific attention. Knowledge gained from dietary research can help anticipate ecological effects [14] and prevent unexpected social and ecological outcomes that could compromise coexistence with humans and, ultimately, conservation objectives. The goal of this work is to begin to fill a substantial void in gray wolf dietary research in California via scat analysis. The work presented here is the first investigation of gray wolf diet in the State to use mitochondrial sequencing for carnivore species confirmation and microsatellite genotyping for individual wolf identification, combined with DNA metabarcoding to determine prey species in scat samples. This study was conducted in a heavily human-modified landscape with high livestock density and limited wild ungulate abundance and diversity with the objective of describing and quantifying gray wolf diet composition. This research will aid scientists and conservation practitioners in developing a more comprehensive understanding of gray wolf dietary ecology across landscapes with varying levels of anthropogenic modification.

## Methods

### Study area

The study area consisted of the summer wolf pack territories of the Lassen (2022 and 2023) and Harvey (2023) Packs in the Northeastern region of California, USA (4,046 km^2^) [15]. The Lassen Wolf Pack occurred in southern Lassen and northern Plumas Counties, California where it successfully produced litters from 2017 to 2024 [16]. The Lassen Pack’s den sites and most of their rendezvous-site locations were close to large meadow complexes where cattle grazing occurs throughout the summer [17]. The Harvey Pack established in northern Lassen County and was confirmed to have produced its first litter in April 2023. The documented distinct summer and winter space use of the Lassen Pack [17], along with the recent establishment of the Harvey Pack in the study area facilitated the collection of scats known to represent the packs’ summer diet.

The territories of the Lassen and Harvey Packs entirely overlap with U.S. Forest Service grazing allotments and private land where cattle are present from May through October each year. Permitted cattle grazing occurs annually across the Eagle Lake Ranger District of the Lassen National Forest from June through October [18]. Grazing allotments range from approximately 40 km^2^ to 121 km^2^ with stocking rates ranging from approximately 600–3,200 animal unit months (cow-calf pairs) per allotment [18]. Privately-owned land in the region is intermixed with U.S. Forest Service allotments and is leased for cattle grazing with temperature and precipitation dictating the duration, commonly from May through October each year. Scat collection was conducted under land access agreements with private timber companies, the Eagle Lake Ranger District of Lassen National Forest, and participating ranch owners and operators.

The Lassen and Harvey Packs established themselves in an area characterized by a traditional western U.S. cow-calf husbandry system consisting of free-ranging cattle herds with access to large grazing areas with limited fencing and cross-fencing and varying levels of surveillance. Range-based cow-calf operations in California exhibit low natural mortality rates (1% for cows and 2% for calves) [19]. The combination of large herd sizes, topographic complexity, and the need for cattle to range widely to access native forage and water collectively render most non-lethal deterrence tools impractical to implement at a meaningful scale. On Forest Service allotments and private timber company leases, non-lethal deterrence is further constrained by permitting and approval requirements, the absence of night penning facilities, and liability and insurance barriers to placing range riders or guardians on the land.

Carcass disposal practices varied across the study area. Where feasible, carcasses were removed and buried or transported to a rendering facility; however, carcasses discovered in remote locations or involving animals too large to move were left in place and consumed by wildlife.

### Sample collection

We collected scats opportunistically from June to October of 2022 and 2023 along dirt roads and game/livestock trails throughout the study area coinciding with other wolf and livestock monitoring efforts and fieldwork. To maximize the number of wolf scats collected and avoid biases by age (e.g., overlooking smaller scats from pups), we collected all carnivore scats potentially from a wolf. Wolf GPS collar data were not available; therefore, scats were not collected from den sites, rendezvous sites, or GPS cluster locations. Collected scats were stabilized with desiccant until processing.

### Species typing and genotyping

All laboratory procedures were conducted at the Mammalian Ecology and Conservation Unit of the Veterinary Genetics Laboratory at the University of California, Davis, following standard procedures for which details have been published previously (e.g., [20–24]). Briefly, genomic DNA was extracted from a 200 mg subsample of each scat using the GeneMatrix Stool Purification kit (EURx, Ltd., Gdansk, Poland), following the manufacturer’s instructions, except that DNA was eluted into 50 µl instead of 200 µl of buffer to increase DNA concentration [20]. Host species was identified by Sanger sequencing a fragment of the mitochondrial cytochrome *b* gene using highly conserved vertebrate primers RF14724 and RF15149 [21], which produce an amplified sequence that was directly comparable to sequences from known species. Polymerase chain reactions (PCR) and Sanger sequencing steps were performed as previously described and compared to an in-house database of vertebrate sequences [22]. The samples identified as wolf were then genotyped using 13 microsatellite loci and a sex marker (SRY) as described in detail previously [24].

### Diet metabarcoding

Diet metabarcoding procedures, including primer sequences, also followed standardized protocols of the Mammalian Ecology and Conservation Unit, and have been described in detail elsewhere [23,24]. Briefly, we included replicates of a subset of samples and control samples (including no fecal DNA, but all other reagents), including positive controls that had DNA from known species, for quality control in data analysis. Libraries were constructed in two PCR steps, amplicon and index PCRs. The amplicon PCR amplified the target region of the 12S gene of the vertebrate mitochondrial genome, whereas the index PCR attached unique 6–8 bp index combinations to each sample’s PCR product along with Illumina adapters enabling sequencing. The primers used in the amplicon PCR included a blocking primer that reduced amplification of wolf DNA. The amplicon PCRs were performed in 11-µl volumes containing 1x Qiagen Multiplex Mastermix (Qiagen, Valencia, CA), 1x Q-solution (Qiagen), 0.8 uM blocker oligonucleotide, 0.08uM P5-12SV5 primer, 0.16 uM P7-12SV5 primer, and 2 µl of fecal DNA. The thermal profile included a 15-minute denaturation step at 95 ˚C, followed by 32 cycles of 94 ˚C for 30 seconds and 55 ˚C for 90 seconds (and retained at 15 ˚C). The amplicon PCR product was then used as the template for the index PCR, which were performed in 25.5 µl reaction volumes containing 2x NEBNext Ultra II Q5 Master Mix (New England Biolabs), 1 uM each of custom i5 and i7 indexed primers, and 3 µl of cleaned amplicon PCR product. The thermal profile included a 30 second denaturation step at 98˚C, followed by 8 cycles of 98˚C for 10 second and 65˚C for 75 second, followed by 65˚C for 5 minutes (and retained at 4˚C). Amplification products were then pooled and sequenced on an Illumina Novaseq X platform (Admera Health, South Plainfield, NJ) separately for 2022 and 2023 batches.

Bioinformatic methods followed a standard pipeline [23,24]. Sequencing reads were sorted (demultiplexed) into sample-specific files containing raw sequencing reads and then trimmed of adapter sequence using cutadapt [25]. Next, trimmed reads were sorted into amplicon sequence variants (ASVs) in DADA2 [26], producing a table with the numbers of reads corresponding to each ASV in each sample (hereafter, “occurrences”). We then use the Basic Local Alignment Search Tool (BLAST) in the nucleotide database of NCBI to match ASVs to species or the most specific taxonomic grouping possible. When multiple ASVs corresponded to the same species or most finely resolved taxon, they were merged. Taxonomic ambiguities were often resolved based on which species ranges overlapped the study areas. Unexpected attributions (e.g., chicken, pig) could have indicated anthropogenic sources in some cases and were therefore retained. Valid dietary items were tabulated in terms of the top BLAST hit and percentage sequence identity. Once dietary items were provisionally assigned to a species or other taxonomic level, an unfiltered (i.e., raw) dataset was compiled. We removed reads corresponding to the host species (i.e., wolf) or those that were rare and indicative of human contamination, parasites, nuclear mitochondrial insertions (numts), or unknown species with no BLAST result matching above 95%. We assessed agreement between replicates in terms of the correlations between numbers of reads assigned to each dietary species and then merged replicates for analysis by averaging read counts for each occurrence within a scat. We used negative and positive control samples to assess biases due to background contamination (e.g., barcode hopping during sequencing), which can result in small numbers of contaminant reads assigned to samples [23,24]. Where warranted, we employed minimum thresholds for numbers of read counts, specifically, the maximum number of contaminant reads from any dietary species detected in any control sample from that sequencing run (S1 Appendix). Data were then summarized as frequency of occurrence, calculated by counting the number of scats in which each prey item was detected (e.g., above a minimum threshold number of reads) and dividing by the total number of scats assessed. We also computed 95% confidence intervals for frequencies of major dietary groups based on the relationship between the *F* and binomial distributions [27].

### Biomass by prey species

Biomass estimation is widely used to account for variation in prey body size and considered by some in the existing literature to be the best approximation of true diet [28–30]. Although frequency-of-occurrence measures facilitate a relative comparison of diet in space and time, they fail to quantify the biomass of a particular prey that is consumed [29,31]. To estimate biomass consumption, we used the linear relationship developed by Floyd [32] and refined by Weaver [33] specific to gray wolves that connects the average live mass (kg) of a prey species *i,* denoted $X_{i}$ to the mass of that prey consumed (kg) as represented by its occurrence in the scat:


$$\begin{array}{c} \hat{Y_{i}}=0.439+0.008X_{i} \end{array}$$ (1)

To facilitate comparison with other wolf diet studies (e.g., Davis et al. [34], Gable et al. [35], and Janerio-Otero et al. [31]), we then calculated the percent biomass, $B_{i},$ for each prey species *i*:


$$\begin{array}{c} B_{i}=\frac{n_{i}⬝\hat{Y_{i}}}{\sum_{i=1}^{j}n_{j\;}⬝\hat{Y_{j}}} \end{array}$$ (2)

where $n_{i}\;$ is the number of scats containing prey species *i.* The denominator of (2) (i.e., total biomass) is the sum of biomass attributions ($n_{i}⬝\hat{Y_{i}}$) across all prey species *i* = 1,…*j*.

Live mass (kg) for each identified prey species is provided in Tables 1 and 2 for 2022 and 2023, respectively. When mass ranges were available for adult prey species, the median of the lower and upper values was used [36]. When more information was available specific to the study area and prey species, live masses were chosen to more accurately reflect typical prey as recommended by Floyd et al. [32].

**Table 1:** Species of diet item, category, number (No.) of scats, frequency of occurrence (FOO), live mass, and biomass percent for diet items identified via metabarcoding in 56 scat samples in 2022.

Diet Item	Category	No. Scats	FOO (%)	Live Prey Mass (kg)	% Biomass
Cattle	Cattle	48	85.7	181.50	60.2
Mule deer	Mule deer	21	37.5	17.15	8.0
Pocket gopher^a^	Small mammal	13	23.2	0.19	3.8
Montane vole	Small mammal	12	21.4	0.06	3.5
Botta’s pocket gopher	Small mammal	8	14.3	0.19	2.3
Black bear	Black bear	5	8.9	8.06	1.7
Sooty grouse	Avian	4	7.1	1.03	1.2
Mountain quail	Avian	4	7.1	0.24	1.2
Chicken	Avian	4	7.1	0.12	1.2
Striped skunk	Other	4	7.1	3.18	1.2
Chipmunk (unknown)	Small mammal	3	5.4	0.07	0.9
California ground squirrel	Med mammal	3	5.4	0.51	0.9
Meadow vole	Small mammal	3	5.4	0.06	0.9
Deer mouse	Small mammal	3	5.4	0.04	0.9
Bison	Bison	3	5.4	91.0	2.3
Pied-billed grebe	Avian	3	5.4	0.41	0.9
American pika	Med mammal	2	3.6	0.15	0.6
Finch (unknown)	Avian	2	3.6	0.02	0.6
Golden-mantled ground squirrel	Med mammal	2	3.6	0.46	0.6
Snowshoe hare	Med mammal	2	3.6	1.59	0.6
Cougar	Other	2	3.6	47.63	1.1
Water vole	Small mammal	2	3.6	0.06	0.6
Passerine (unknown)	Avian	1	1.8	0.03	0.3
American bullfrog	Other	1	1.8	0.50	0.3
Brown bullhead	Other	1	1.8	0.68	0.3
California pocket mouse	Small mammal	1	1.8	0.02	0.3
Horned lark	Avian	1	1.8	0.03	0.3
House mouse	Small mammal	1	1.8	0.02	0.3
Shrew (unknown)	Small mammal	1	1.8	0.01	0.3
Western spotted skunk	Other	1	1.8	0.68	0.3
Bobcat	Other	1	1.8	8.39	0.3
Black-necked grebe	Avian	1	1.8	0.4	0.3
Thrush (unknown)	Avian	1	1.8	0.05	0.3
White fronted goose	Avian	1	1.8	2.25	0.3
Myotis (unknown)	Other	1	1.8	0.04	0.3
Great Basin pocket mouse	Small mammal	1	1.8	0.02	0.3
Belding’s ground squirrel	Med mammal	1	1.8	0.29	0.3
Pig	Other	1	1.8	0.12	0.3
Tanager (unknown)	Avian	1	1.8	0.03	0.3

^a^ Mountain or northern pocket gopher.

**Table 2:** Species of diet item, category, number (No.) of scats, frequency of occurrence (FOO), live mass, and biomass percent for diet items identified via metabarcoding in 42 scat samples in 2023.

Diet Item	Category	No. Scats	FOO (%)	Live Prey Mass (kg)	% Biomass
Cattle	Cattle	23	54.8%	181.50	50.3
Mule deer	Mule deer	23	54.8%	17.15	15.2
Montane vole	Small mammal	19	45.2%	0.06	9.6
Turkey	Avian	6	14.2%	4.54	3.3
Botta’s pocket gopher	Small mammal	4	9.5%	0.19	2.0
Cougar	Other	4	9.5%	47.63	3.8
Passerine (unknown)^a^	Avian	3	9.5%	0.03	1.5
Sparrow (Passerellidae)	Avian	3	7.1%	0.03	1.5
Water vole	Small mammal	3	7.1%	0.06	1.5
Belding’s ground squirrel^a^	Med mammal	3	7.1%	0.29	1.5
Golden-mantled ground squirrel	Med mammal	2	4.8%	0.26	1.0
Black bear	Black bear	2	4.8%	8.05	1.2
Pocket gopher^b^	Small mammal	2	4.8%	0.19	1.0
California ground squirrel	Med mammal	2	4.8%	0.51	1.0
Duck (Anatidae)	Avian	2	4.8%	1.15	1.0
Shrew (unknown)^a^	small mammal	2	4.8%	0.01	1.0
Black-tailed jackrabbit	Med mammal	1	2.4%	2.05	0.5
Great basin pocket mouse	Small mammal	1	2.4%	0.02	0.5
Dark-eyed junco	Avian	1	2.4%	0.02	0.5
Douglass squirrel	Med mammal	1	2.4%	0.23	0.5
Horned lark	Avian	1	2.4%	0.03	0.5
Mountain quail^a^	Avian	1	2.4%	0.24	0.5
Deer mouse^a^	Small mammal	1	2.4%	0.04	0.5
Chipmunk (unknown) ^a^	Small mammal	1	2.4%	0.07	0.5

^a^ Six dietary items were not subject to a minimum read filter; all others were required to exceed 181 reads.

^b^ Mountain or northern pocket gopher.

Calves ranging from 136–227 kg are the most frequent size of cattle that are confirmed to be depredated by wolves in California during the study period [37]. As such, we use the median of this weight range as our estimate of average live prey mass for cattle. During the study period, USDA Wildlife Services personnel confirmed wolf predation on full-sized cows (544–680 kg) within the study area [37]. Using calf mass in the biomass calculations will make our results conservative when attributing dietary prey volume to livestock, likely understating its actual contribution to biomass percentage and wolf diet composition. To bound the range of reasonable biomass estimates, we conducted a sensitivity analysis in which bovine mass was allowed to vary from 136 kg — the smallest calf confirmed depredated during the study period — to 680 kg, representative of a full-sized commercial cow in the study area.

Game cameras maintained in the study area confirm that wolves consumed young fawns during the summer season when scat was collected. The median value of a newborn fawn mass (2.3 kg) and the mass of a female doe at 6 months of age (32 kg) was used to represent the average live prey mass for mule deer (*Odocoileus hemionus*). Similarly black bear (*Ursus americanus)* cubs are subject to wolf predation; the median of average cub mass when born (0.23 kg) and average mass at 6 months of age (15.88 kg) was used to represent live prey mass.

Several irregular diet items, including bison, chicken (*Gallus gallus domesticus)*, and pig (*Sus scrofa domesticus)*, were detected in the scat samples. A portion of the Lassen Pack’s territory includes an agricultural operation that raises and slaughters bison; this entity disposes of offal post slaughter by feeding it to wildlife. Thus, in the biomass calculations the average mass of bison offal (91 kg) is used. Both the chicken and pig detected in scat samples emanated from anthropomorphic waste disposal sites. Ascribed live prey masses are based on the likely negligible amounts that remained post disposal; 0.12 kg for an average medium sized chicken breast or average sized pork chop.

### Ungulate biomass availability

Although we had no data on biomass availability of small and medium prey species, wolf diet tends to be disproportionately dependent on larger-bodied prey, principally ungulates. Therefore, we estimated the relative availability of deer and cattle biomass present within the summer home range of the Lassen Pack in 2022 and the combined summer home ranges of both packs in 2023. The size of wolf territories typically remains the same from year to year. Therefore, we used the only publicly available data to estimate the Lassen and Harvey pack territories at approximately 1,100 km² and 1,700 km², respectively, by digitizing pack boundaries from the CDFW January 2024 wolf activity map using image-based georeferencing.

The most recent mule deer density estimates available for the study area are derived from Furnas et al. [38], based on data collected during 2015–2016, which yielded an estimate of 5.2 deer per km² with a demographic ratio of 1.0:0.37:0.67 in terms of female:male:fawn. Using weighted average live weights of 20 kg for fawns, 54 kg for females, and 65 kg for males, deer biomass within the Lassen Pack home range of 256,301 kg and within the combined Lassen and Harvey pack areas totaled 652,403 kg.

Using stocking data from U.S. Forest Service (USFS) allotments, timber company grazing leases, and private lands, we estimated cattle numbers and biomass within the packs’ territories. For USFS allotments, the number of cow-calf pairs for 2022 and 2023 for each allotment was determined by the Annual Operating Instructions provided to the permittee by the USFS. Because private timber lands are intermixed with USFS allotments and fencing or other physical boundaries between these land tenures are uncommon, cattle effectively range across both in an integrated grazing system. A small portion of the study area consists of privately owned, seasonally irrigated native meadow, which supports substantially higher forage productivity and stocking densities (50–200 head/km^2^) than unimproved upland Forest Service allotments or timber leases (0.6–1.7 head/km^2^). Cattle numbers and biomass on these irrigated meadows were estimated separately to account for differences in carrying capacity, and producers were queried to ensure that cattle were not double counted across private land and USFS allotment records. Using the average live weights for cows of 544 kg and calves of 249 kg results in an available cattle biomass estimate of approximately 3.2 million kg in the Lassen Pack home range and approximately 4.0 million kg in the combined pack areas.

## Results

Over two years, a total of 384 scat samples were collected. DNA genotyping confirmed 105 samples (27%) as gray wolf, 245 (64%) as coyote (*Canis latrans)*, 6 (<2%) as bobcat (*Lynx rufus)*, 6 (<2%) as black bear (*Ursus americanus)*, 1 (<1%) as mountain lion (*Puma concolor*), and 21 (5%) failed sequencing.

After species typing, microsatellite genotyping was used to identify individual animals and determine the sex of those individuals for all scats that were confirmed to be wolf (n = 105). Of the 59 total wolf scats collected in 2022, 47 samples (80%) were successfully microsatellite genotyped, identifying 13 individual wolves – 6 females and 7 males. In 2023, 34 (74%) of the 46 wolf scats were successfully microsatellite genotyped, identifying 13 individuals (5 males, 8 females). In total, we identified 20 individual wolves (8 M, 12 F) from 79 of the 105 wolf scats for which we were able to obtain successful genotypes, including samples from six individual animals in both years.

### Wolf diet composition

We obtained dietary data from 98 of the 105 wolf scats attempted, including 77 of the 81 scats that successfully genotyped. The correlation of read counts per dietary item was high between replicates (*r* = 0.972, n = 58 pairs). After filtering, we identified 45 dietary items in the two years combined, of which 18 were observed in both years (Tables 1, 2). Scat samples from 2022 and 2023 were represented, respectively, by 30% and 21% containing one diet item, 16% and 24% containing two diet items, and 54% and 55% containing three or more diet items. Taxa other than cattle, mule deer, and black bear were grouped into the following categories: small mammal, medium mammal, avian, and other for downstream analyses (Tables 1, 2).

**Fig 1:**
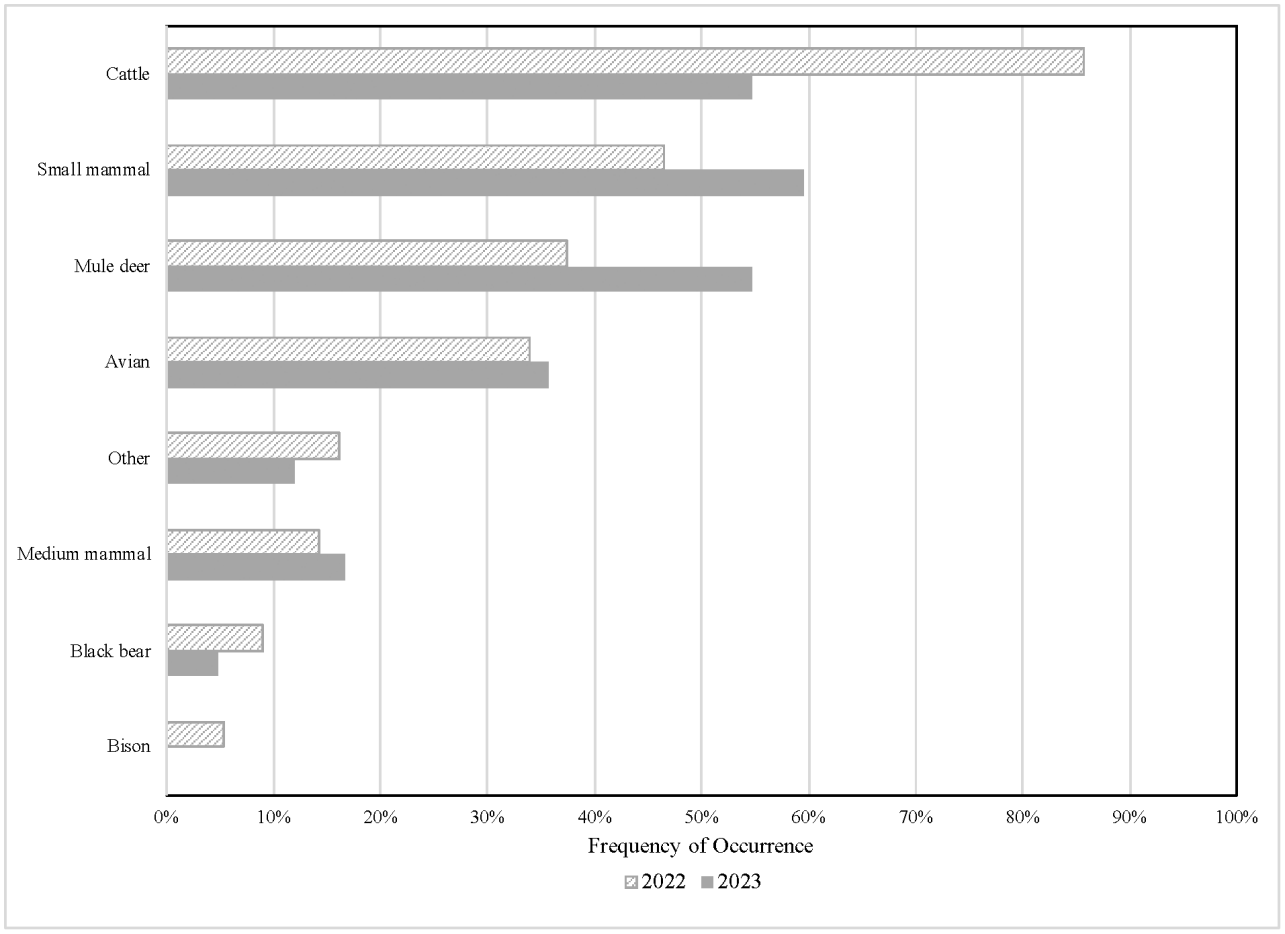
Gray wolf diet in terms of frequency of occurrence for summers of 2022 and 2023.

Livestock, specifically cattle, were a frequently occurring diet item during both summers of the study, present in 86% and 55% of scats collected in 2022 and 2023, respectively (Fig 1). While all scats in 2022 were from the Lassen Pack, the 2023 samples included 8 from the Harvey Pack, which, while too small a sample for an independent estimate of diet, also showed significant consumption of cattle and deer. During 2022, 45% of the samples contained small mammals and 38% of the samples contained mule deer. In 2023, 60% of the samples contained small mammals and 55% of the samples contained mule deer. Across the combined dataset, cattle were detected in 72% of 98 scats (95% CI: 63–81%), which was significantly more frequently than mule deer, which were detected in 45% of the same scats (95% CI: 35–55%; Chi-Square, 1 df = 15.3, P << 0.001).

**Fig 2:**
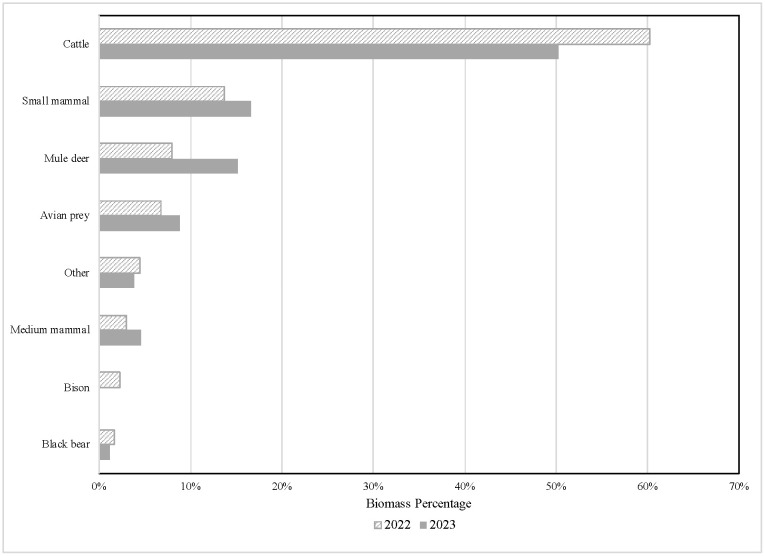
Gray wolf diet in terms of biomass percentage for summers of 2022 and 2023.

Consistent with frequency-of-occurrence, estimates of the composition of the wolf summer diet based on prey biomass showed that cattle were a significant component of the diet, accounting for 60% and 50% of the biomass in 2022 and 2023, respectively (Fig 2). The biomass contribution of mule deer to wolf diet, however, was estimated at only 8% and 15% of biomass in 2022 and 2023, respectively. Biomass consumption estimates for small mammals were similar to those of deer in both years: 14% in 2022 and 17% in 2023. The proportional contribution of cattle with respect to the two main ungulate diet items (deer, cattle) was 88% in 2022 and 78% in 2023.

Sensitivity analysis in which bovine mass was varied from 136 kg to 680 kg produced a range of estimated cattle biomass contributions to wolf diet. Using the minimum mass of 136 kg, cattle accounted for 55% of total estimated biomass consumed in 2022 and 51% in 2023. When bovine mass was set at the maximum value of 680 kg, representative of a full-sized commercial cow, cattle’s estimated contribution to total biomass consumed increased to 82% in 2022 and 80% in 2023. During the 2017–2019 period, Dellinger et al. [39] derived bovine mass from the average estimated live weight of calves, yearlings, and cows present in the Lassen Pack’s home range of 272 kg. Using this estimate, cattle’s estimated contribution to wolf biomass consumed was 68% in 2022 and 65% in 2023. These results indicate that the proportional contribution of cattle to wolf diet was sensitive to the assumed bovine mass, and that the use of calf mass (181.5 kg) in the primary analysis produced a conservative estimate (60% and 50% in 2022 and 2023, respectively) of cattle’s true contribution to wolf diet in the study area.

We obtained dietary data for 77 of the individually identified scats the two years of the study, including at least one scat from 20 individual wolves: a male and female from the incipient Harvey Pack, along with 7 males and 11 females from the Lassen Pack. At least 17 (85%) of the wolves had consumed cattle; the three individuals for which we did not document consumption of cattle were each represented by a single scat.

### Ungulate biomass availability

Despite the high consumption of cattle among ungulate diet items suggested by our dietary analysis (88% in 2022, 78% in 2023), consumption of cattle was slightly lower than expected based on availability, which we estimated at 93% of the prey biomass available in 2022 and 86% in 2023. In contrast, deer composed a higher proportion of ungulate biomass consumed (12% in 2022, 22% in 2023) than expected based on availability, which we estimated at 7% of the prey biomass available in 2022 and 14% in 2023.

## Discussion

Regardless of the metric employed – frequency of occurrence and dietary biomass – estimates during our study consistently indicated that cattle were the predominant dietary item of wolves in Northeastern California during the summer months. Given that both dietary summary metrics (frequency of occurrence and dietary biomass) possess established biases, their combined use is considered to yield the most complete characterization of diet [28,40]. The only other gray wolf diet study conducted in California to date yielded similar results. Dellinger et al. [17], who employed scat diameter for species identification and macroscopic hair analysis for prey determination, reported lower frequency of occurrence (32%) but comparable biomass percentage (59%) estimates for cattle in 2017–2019. These collective findings differ markedly from research conducted in other areas in North America. Newsome et al.’s [13] metanalysis indicated that in North America gray wolf diet is dominated by large- and medium- sized ungulates, particularly mule deer, elk (*Cervus canadensis*), white-tailed deer (*Odocoileus virginianus*), moose (*Alces alces*), and caribou (*Rangifer tarandus*). Further, Newsome et al. [13] reported that domestic species (including livestock) were only present in 10 studies in North America and, when they were detected, domestic species only comprised 8% of gray wolf diet. Several important factors account for this difference in diet composition.

The first, and somewhat obvious, reason is that California lacks the total quantity and diversity of large- and medium-sized wild ungulates present in other parts of North America where gray wolf diet has been studied. White-tailed deer, moose, and caribou do not occur in California. While California is home to elk, their numbers are relatively low in the state as a whole and in Northeastern California, in particular [41]. The extent of documented area inhabited by elk does not include the majority the Lassen Pack’s home range. There is substantial overlap of elk range and Harvey Pack home range. In California’s Wolf Management Plan [41], concern was expressed about the ability of the elk population to handle predation from wolves: “Most of California’s elk populations are small, which creates the possibility of localized extirpation…”. Given that prey density and overall prey abundance are documented to be drivers of gray wolf diet [42], it is not surprising that elk was not observed to contribute to the summer diet of the Lassen or Harvey Packs.

Second, the only viable wild ungulate prey in the State, mule deer, has been in population decline since the 1970s [41]. On a regional level, deer populations in Northeastern California have experienced the sharpest percentage declines in the State – from approximately 950,000 in 1990–250,000 in 1996 [43]. The most recent population estimate (2017) available from California Department of Fish and Wildlife is 532,621 mule deer in the State. While a myriad of factors – including habitat conversion, fire, urbanization, road traffic, and increasing predator populations – likely play a role in deer population decline, depletion of the key wild ungulate prey available for wolves may be one factor influencing gray wolf diet selection in California.

Irrespective of underlying causes, the substantial cattle consumption by wolves documented in both our study and previous research in this region [14] underscores the complexities associated with wolf recolonization of California’s contemporary landscape. As with other scat-based dietary studies, our methods cannot differentiate predation from scavenging; thus, these results do not imply that wolves killed the entirety of the cattle they consumed nor do they indicate what proportion could be attributed to predation versus scavenging. Further investigation into wolf foraging ecology in California would be beneficial. Nevertheless, wolves are known to predate cattle, and range-based cow-calf operations in California typically exhibit low natural mortality rates (1% for cows and 2% for calves) [19]; therefore, it is reasonable to expect that a meaningful portion of the cattle biomass detected in wolf diet reflects predation rather than scavenging alone. USDA Wildlife Services depredation investigations confirmed a minimum of seven cattle depredations attributable to the Lassen and Harvey packs during the study period [44]. Further, analysis of CDFW depredation records for the period 2017–2024 indicates average annual depredation rates of 3.5 events per year for the Lassen Pack and 4.5 events per year for the Harvey Pack [44]. While we cannot confirm predation directly, our findings indicate that cattle constitute a significant portion of the energetic requirements of wolves in this area.

A limitation of this study is its focus on two packs in California. However, USDA Wildlife Services and CDFW depredation reports document considerably higher cattle predation rates in areas occupied by other wolf packs in the state. The Lassen and Harvey packs in 2022 and 2023 had seven confirmed cattle depredations; over the same period the Whaleback pack had 49 confirmed depredations [4]. While further investigation into the causes for these disparities – including differences in access to staff for depredation investigations and native ungulate abundance – is warranted, the elevated cattle depredation rates documented for other packs suggest our findings may be broadly relevant to existing packs statewide and highlight the complexities of coexistence during wolf recolonization of California’s contemporary landscape.

## Supporting information

S1 Appendix Quality control and filtering. (PDF)
