# Supplementary material for: Gray Wolf Diet Composition in California’s Human-Dominated Landscape
Source: PLoS One. 2026 Jul 8;21(7):e0351768. doi: 10.1371/journal.pone.0351768 (PMC13345248; doi:10.1371/journal.pone.0351768)
Supplement: S1 Appendix — (PDF) [file pone.0351768.s001.pdf]

S1 Appendix: Quality control and filtering

The amount of sequencing differed in the two years, with the average number of sequencing reads produced per sample approximately 30x higher in 2023 (90,301 dietary reads) than in 2022 (3,420 dietary reads). Therefore, we assessed and filtered for background contamination separately in the two years based on 20 negative controls and 14 positive controls in 2022, and 20 negative controls and 20 positive controls in 2023.

In the 2022 dataset, we found very little background contamination based on the ratio of contaminant read counts in controls to samples (0.002), as well as the proportion of reads that were contaminants in the positive control sample (average = 0.06%, maximum = 0.23%, n = 14 positive controls), and, lastly, on the average total number of contaminant dietary reads (n = 10) in controls (positive and negative); additionally, 23 of 34 controls had no contaminant reads and 11 had only 2–133 reads each of one of 6 dietary species. We observed no contaminant reads of either cattle or deer in any control. Because risk of false positives was low, we considered any occurrence of reads from a dietary item in a scat sample to represent true presence of that dietary item (i.e., we did not employ a minimum-read threshold). Cattle occurrences in wolf scat samples were represented by an average of 2,076 (range = 11 to 28,989) reads each and those of deer were represented by an average of 1,726 (range = 6 to 23,424) reads each. Had we employed a minimum threshold for read counts in this dataset (i.e., 133 reads), the frequencies of cattle and deer would have declined (i.e., presumably been underestimated). The average number of dietary items per scat also would have declined from 3.0 to 1.7, including loss of 19 dietary items entirely from the dataset; these items were especially unlikely to represent background contamination as there was no other likely source.

In 2023, we observed a similarly low contamination fraction, for example, proportion of reads that were contaminants in positive controls averaged 0.079% (maximum = 0.21%; n = 20 positive controls). However, the much higher volume of sequencing performed on samples in 2023 resulted in low but consistent levels of background contamination (average total No. dietary reads = 92); 40 control samples (negative and positive) showed 12–394 contaminant dietary reads each among 25 dietary items. Contaminant species included cattle, for which the maximum number of reads in any control was 55, and deer, for which the maximum number of reads in any control was 70. The highest number of contaminant reads attributed to any dietary item (Montane vole) was 181 reads, which we used as a minimum threshold to count occurrences in samples. After filtering, the number of dietary items per scat averaged 2.8 which was similar to the 3.0 number of dietary items observed per scat in 2022 (which was not subjected to a threshold filter). During filtering, however, there was a loss of 6 rare dietary items from the dataset. Because these rare occurrences were unlikely to reflect contamination (i.e., because they were rare), we used the filter for all other dietary items but included these rare items despite their not exceeding the threshold.
